# Effect of increased prolactin and psychosocial stress on erectile function

**DOI:** 10.1192/j.eurpsy.2022.715

**Published:** 2022-09-01

**Authors:** R. Sajdlova, L. Fiala

**Affiliations:** University Hospital in Pislen, Psychiatry Department, Pilsen, Czech Republic

**Keywords:** erectile dysfunction, Hyperprolactinemia, psychosocial stress

## Abstract

**Introduction:**

Sexual dysfunctions in men are complex disorders that consist of organic and psychogenic components. The most common sexual dysfunction is erectile dysfunction. It is the inability to achieve or maintain an erection for satisfactory sexual performance. This disorder can be caused by high blood pressure, heart disease, vascular problems, psychological and hormonal factors such as problems with testosterone and prolactin levels.

**Objectives:**

The most common sexual dysfunction is erectile dysfunction. It usually affects men over the age of 40. The causes of erectile dysfunction can be organic, psychogenic or a combination of both. The most common organic causes of erectile dysfunction may be high blood pressure, diabetes mellitus, obesity or hormonal disorders. Psychogenic reasons are usually related to psychosocial stress. In this study, we tested the relationship between erectile dysfunction, hyperprolactinemia, and psychosocial stress.

**Methods:**

Clinical examinations of 60 patients with erectile dysfunction, which also included psychosocial stress, focused on patient history, comprehensive sexological examination, biochemical analyzes of serum prolactin, total testosterone, thyroid stimulating hormone with psychometric evaluation of erectile function and a checklist of trauma symptoms (TSC-40)

**Results:**

The results show significant Spearman correlations of psychometric evaluation of erectile function with prolactin (R = 0.50) and results of the trauma checklist score (R = 0.55) as well as significant Spearman correlations between TSC-40 and prolactin (R = 0.52). This result indicates a significant relationship between erectile dysfunction, hyperprolactinemia and stress symptoms in men.

**Conclusions:**

Our result indicates a significant relationship between erectile dysfunction, hyperprolactinemia and stress symptoms in men.

**Disclosure:**

No significant relationships.

